# Improving preoperative prediction of surgery duration

**DOI:** 10.1186/s12913-023-10264-6

**Published:** 2023-12-02

**Authors:** Vahid Riahi, Hamed Hassanzadeh, Sankalp Khanna, Justin Boyle, Faraz Syed, Barbara Biki, Ellen Borkwood, Lianne Sweeney

**Affiliations:** 1 The Australian e-Health Research Centre, Commonwealth Scientific and Industrial Research Organisation, Melbourne, VIC Australia; 2 The Australian e-Health Research Centre, Commonwealth Scientific and Industrial Research Organisation, Brisbane, QLD Australia; 3 Fiona Stanley Hospital, Western Australia Health, Perth, WA Australia

**Keywords:** Operating room, Operation duration, Prediction models, Individual patient characteristics, Extreme gradient boosting

## Abstract

**Background:**

Operating rooms (ORs) are one of the costliest units in a hospital, therefore the cumulative consequences of any kind of inefficiency in OR management lead to a significant loss of revenue for the hospital, staff dissatisfaction, and patient care disruption. One of the possible solutions to improving OR efficiency is knowing a reliable estimate of the duration of operations. The literature suggests that the current methods used in hospitals, e.g., a surgeon’s estimate for the given surgery or taking the average of only five previous records of the same procedure, have room for improvement.

**Methods:**

We used over 4 years of elective surgery records (*n* = 52,171) from one of the major metropolitan hospitals in Australia. We developed robust Machine Learning (ML) approaches to provide a more accurate prediction of operation duration, especially in the absence of surgeon’s estimation. Individual patient characteristics and historic surgery information attributed to medical records were used to train predictive models. A wide range of algorithms such as Extreme Gradient Boosting (XGBoost) and Random Forest (RF) were tested for predicting operation duration.

**Results:**

The results show that the XGBoost model provided statistically significantly less error than other compared ML models. The XGBoost model also reduced the total absolute error by 6854 min (i.e., about 114 h) compared to the current hospital methods.

**Conclusion:**

The results indicate the potential of using ML methods for reaching a more accurate estimation of operation duration compared to current methods used in the hospital. In addition, using a set of realistic features in the ML models that are available at the point of OR scheduling enabled the potential deployment of the proposed approach.

**Supplementary Information:**

The online version contains supplementary material available at 10.1186/s12913-023-10264-6.

## Introduction

The operating room (OR) is one of the pivotal units and valuable assets in hospitals. ORs contribute to about 42% of the revenue generated by a hospital while consuming over 30% of the total hospital costs [1, 2]. The OR administration is a complicated process and ORs’ mismanagement can affect hospital costs and care delivery at different levels, e.g., surgery cancellations [3], nursing staff turnovers due to conflict planning [4], and also staff overtime work and dissatisfaction [5]. Also from a patient’s standpoint, scheduling inefficiencies increase the waiting time for surgery and cause stress and anxiety as a result of surgery cancellations [6]. Therefore, optimising OR efficiency is a priority for many hospitals.

One aspect that helps to optimise OR usage is having a more precise booking of OR schedules [7, 8]. It has been shown that accurate estimation of operation durations may result in a higher efficiency of ORs [9, 10]. However, the OR schedules usually rely on simple methods to estimate operation duration despite their limitations [11, 12]. Such simple methods include using the historical data for a given procedure and then simply calculating an average duration [8], using the surgeon’s estimate for the given operation [8], or taking the average duration of only the most recent records (e.g., previous 5 records) of the same procedure [1, 13]. However, given the considerable variation in operation characteristics, and hence their durations, such simple methods may not result in a reliable estimate of each operation duration and make it even more challenging to derive an accurate OR schedule.

There have been several attempts to provide models for better operation duration prediction. Existing models can be categorised into three main groups with several underlying issues. The first category of models focuses on predicting the duration of the surgical procedure instead of predicting the whole operation time (i.e., the time from patients entering the OR to leaving the room) [8, 10, 14–17]. The main limitation of these approaches is that the surgical procedure duration does not capture the entire operation duration due to the fact that an operation also includes other activities that contribute to the total duration, such as anaesthesia induction time and patient recovery time (i.e., the time from the end of the operation until the patient exits the OR) [18]. Besides, an operation may involve multiple procedures that are not necessarily undertaken sequentially throughout the operation. Another category of models focuses only on a limited number of subspecialties [19–22]. The main challenge of this approach is that, in reality, the clinical administrative team has to manage ORs with a broader range of specialties. Another group of models employ variables that may not be available preoperatively such as the order of an operation in a session, the cancellation status of prior operations, and the time that operations start. As a main drawback, this information may be available in the historical data, however, they are not possibly available at the time of using the models for OR scheduling in a real-world application setting.

In this paper, we focused on providing machine learning (ML) models to predict the operation duration (i.e., “wheels in” to “wheels out”) for each elective surgery, regardless of the number of procedures involved in the operation. We investigated the application of a wide range of ML algorithms to provide a comprehensive evaluation of the ML models for the problem at hand. In order to develop a deployable ML model that can be used to assist administrators at the time of OR scheduling, we restricted the model to those variables that are available preoperatively.

## Materials and methods

### Data sources

The data for this study was extracted from one of the major metropolitan hospitals in Australia. The hospital comprises 783 beds, 15 operating theatres that are available 24 h/day, 5 endoscopy suites, 2 interventional theatres, a dedicated emergency obstetric theatre, 20 preoperative holding bays and 37 post anaesthetic care unit bays. The data was extracted from the Theatre Management System (Department of Health, Government of Western Australia) which is populated exclusively by interfaces with the web-based Patient Administration System (webPAS) and included over 70,000 de-identified records of patients undertaking elective surgeries (i.e., planned surgeries), from November 2014 to June 2020. The study was approved by the FSH QI Medical Anaesthesia & Pain Medicine Committee (Quality activity 29,238) and CSIRO Health and Medical Human Research Ethics Committee (2019_024_LR).

**Fig. 1:**
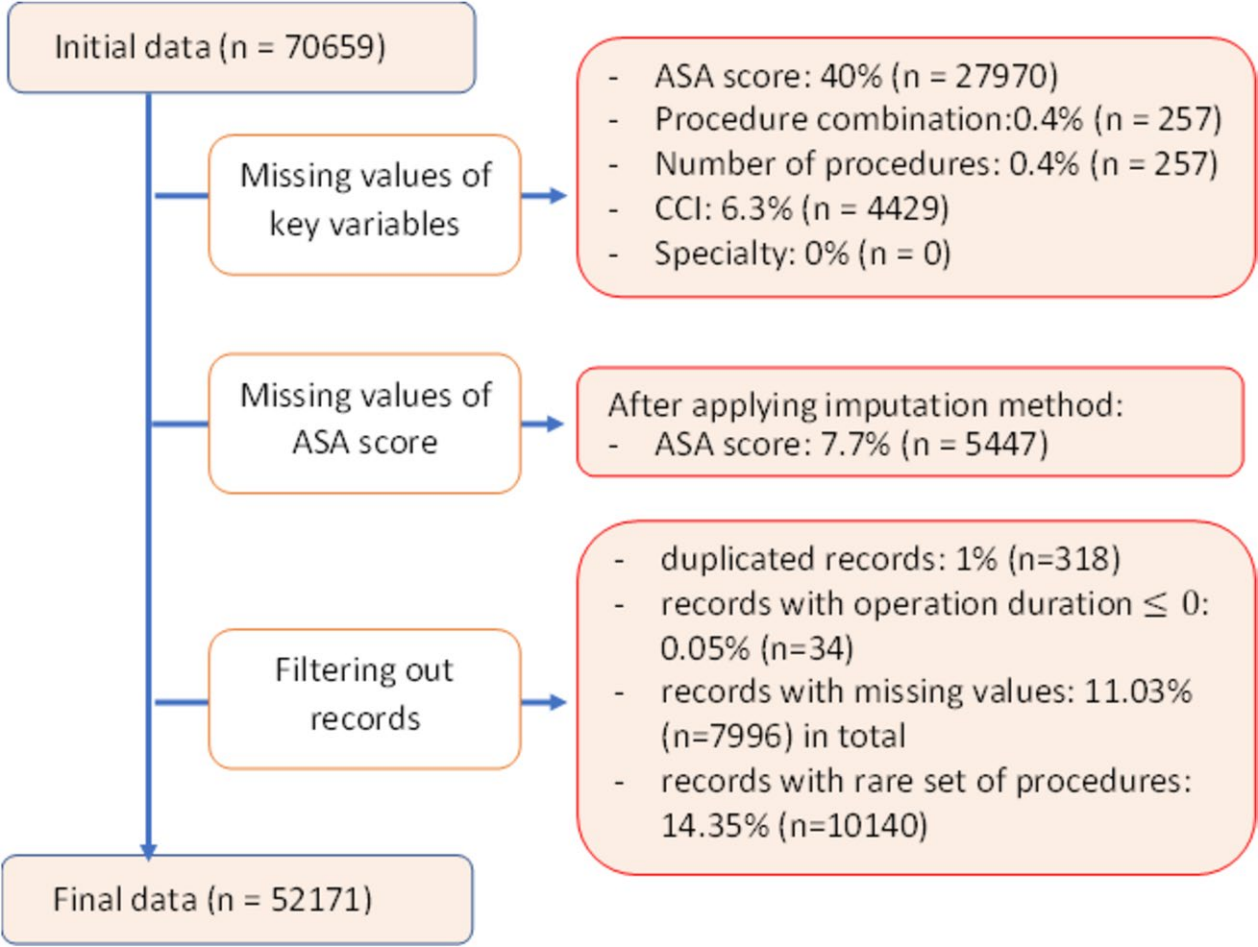
The process of data cleaning with defined conditions

The main aim of this study was to predict the operation duration which is defined as the total minutes from patients entering the OR to patients leaving the OR. To that end, several steps were taken to prepare the data for modelling as follows: (1) operations with multiple records were excluded, (2) surgeries with operation duration recorded as 0 or less were removed, (3) operations with missing values for key variables including specialty and procedures were trimmed, (4) operations that included procedures with less than 10 records within the entire dataset were filtered out. In this study, we assumed that the data is missing at random (MAR). The ASA score (reflecting patient frailty) is one of the candidate variables, however, values were missing for almost 40% of the records. Since the ASA score is a categorical feature, we used the mode formula (i.e., selecting the most frequent value in the dataset) to fill in the missing values [19]. More specifically, for each operation, if it included a missing ASA score, the missing value was replaced by the most frequent ASA score among the previous operations to that date with the same set of procedures. With this method, we reduced the number of records missing the ASA score to only 7.7%. In this study, we conducted a sensitivity analysis to explore alternative scenarios for missing data. We created multiple datasets after applying multiple imputation procedures such as mode, median, last observation comes forward (LOCF) and KNN [23]. In addition, we also created another dataset in which all missing values were filtered out, i.e., only keeping the complete records, and it was confirmed that the results remained similar under alternative strategies. After cleaning the data, 52,171 elective operation records were available for developing predictive models. Figure 1 shows the process of cleaning data and the removed records as a result of each condition.

**Fig. 2:**
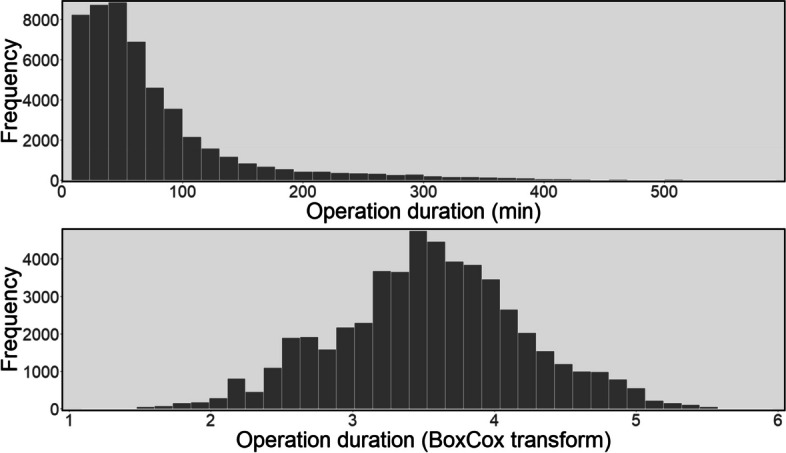
Histogram of operation times for original data (top) and after transformation (bottom)

To evaluate the predictive models we split the data into training and testing data based on the year of operations with the intention of simulating real-world settings where past records are used to predict upcoming events in the future [7, 14, 24–27]. The training dataset included elective operations from November 2014 to June 2019, a total of 41,794 operations, and the testing dataset contained operations from July 2019 to June 2020, a total of 10,377 operations. Also, because of its right skewness, as can be seen in the top plot in Fig. 2, and to improve the accuracy of the prediction models, we applied a BoxCox transformation of the surgery duration times to more closely resemble a normal distribution. As noted in [28], this transformation can be useful for many practical problems including clinical data. It is also worth mentioning that although using log-normal distribution is the most common method to transform operation duration [29, 30], as suggested by [31] we used the BoxCox transformation since log-normal distribution is actually a special case of the BoxCox transformation. Also, the data skewness after log-normal transformation is 0.12, however, the skewness after BoxCox is -0.00062 which means the BoxCox method transforms the operation duration into a more favourable symmetric distribution. Note that, after predicting the duration of surgery, the predicted values were transformed back for accuracy calculations and comparison with the actual values of the datasets. The histogram of the operation times using the given actual data and the transformed version are shown in Fig. 2.

**Fig. 3:**
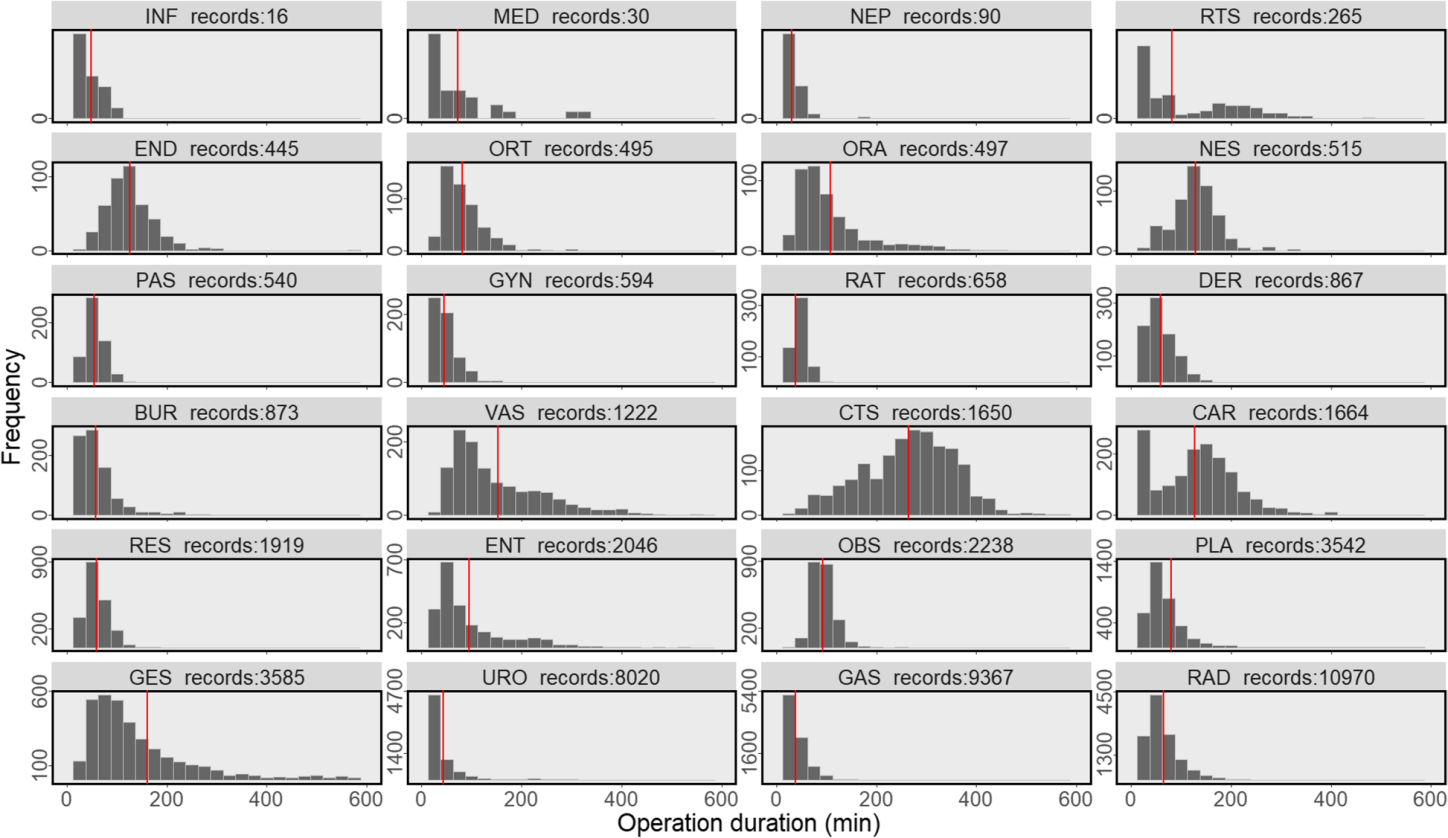
Distribution of the operation times (minutes) for different specialties

Figure 3 shows the distribution of the operation durations (i.e., response variable), across different medical specialties, with at least 15 records. This figure also captured the number of data records contained in each specialty and the average operation time for each specialty, shown by the red colour line.

### Predictor variables for modelling

The predictor variables, used for model development, were selected after an in-depth review of available data sources. First, we focused on variables that are available preoperatively, several days before the operation. Several variables are commonly used in the existing models in the literature and also were available in our dataset including “Order” (e.g., operation order in session), “Session” (e.g., morning or afternoon), “Theatre” (i.e., operating room number), and “Cancellation” (i.e., the cancellation status of the previous operation). However, they were not part of our model development as they would not be relevant in a potential implementation, even though they may improve the model’s accuracy.

Besides the above-mentioned features, we investigated several other common features including “age”, “gender”, “patient history” (i.e., the previous number of surgeries with the same procedures), and “admission type”. But they were also excluded from model development as they either did not improve the model or slightly made the model worse. These findings are consistent with reports from others [14, 32]. It is also worth mentioning that the data used in this study was collected from an OR management database that lacked the information related to the surgeon’s estimation of the operation duration as well as the surgical team (e.g., the surgeon, number of anaesthetists and nurses) was not available. The features used for model development in this study are shown in Table 1. All five predictors were nominal variables.

**Table 1:** Features used for model development

Features	Explanation
Specialty	The primary surgical specialty within the undertaken operation
Number of procedures	Total number of procedures involved in the operation
Procedure combination^a^	The procedures involved in the operation.
CCI	Charlson Comorbidity Index (CCI) [25]
ASA Score	American Society of Anaesthesiologists score is used to determine if someone is healthy enough to tolerate surgery and anaesthesia [26]

^a^Combination of scheduled procedures

The first three features listed in Table 1 (i.e., Specialty, Number of procedures, Procedure combination) are operation-related factors, while the other two are patient characteristics. The CCI is an indicator of the severity and complications of a patient’s condition which uses 17 different patient diagnoses (e.g., diabetes, cancer, liver disease) to calculate the index. Since the calculation of the CCI score needs a thoroughly accurate review of medical records collected over years before hospital admission [33], we considered this feature as it reflects the patient’s medical history. In this paper, the comorbidity package [34] in R was used for computing CCI using the International Classification of Diseases, 10th Revision (ICD-10). It is also worth mentioning that the Present On Admission (POA) diagnoses were used when calculating CCI. The CCI was categorised into three groups: 0, 1–2, and 2+ [35]. The ASA score is a six-level scale, from 1 to 6, that measures the fitness and physical condition of the patient before surgery and anaesthesia. The distribution of the patient characteristics in the given dataset is shown in Table 2.

**Table 2:** Distribution of patient characteristics in the dataset

Features	Range	Percentage of datasets
CCI	0	57
	1–2	26
	2+	17
ASA	1	19
	2	42
	3	33
	4	5
	5	0^a^
	6	0^a^

^a^very close to 0

### Predictive models

Several machine learning (ML) models have been applied to investigate their efficiency and accuracy in predicting surgical operation time: Linear Regression (LR), Polynomial Regression (PR), Random Forest (RF), Extreme Gradient Boosting (XGBoost), and Nearest Neighbours Regression (NNr).

Linear Regression (LR) is a prediction approach that tries to find the best relationship between features (i.e., independent variables) and the response variable by fitting a line to the given data sets [36]. The LR model assigns one scale factor to each feature, called a coefficient, and also one extra coefficient, called the intercept, to give the model an additional degree of freedom. The number of coefficients determines the complexity of the model. Although LR models are easier to implement and interpret, they fail on complex datasets and are quite sensitive to outliers. Polynomial regression (PR) [37] is a form of linear regression. The main difference between PR and LR is that, in PR, the relationship between features and response variable is non-linear (i.e., curvilinear).

Random Forest (RF) [38] is a machine learning algorithm that can be applied for both classification (i.e., the response variable is nominal) and regression (i.e., the response variable is continuous) problems. The RF approach is constructed from decision trees [39] by combining multiple small trees on various subsets of the given dataset to create a ‘forest’. Each tree produces its outputs, and the RF algorithm calculates the average of the outputs of all trees. The main advantage of RF algorithms is that they handle large datasets and also datasets with a great portion of missing values.

Extreme Gradient Boosting (XGBoost) [40] is another ensemble technique based on a decision tree algorithm similar to the RF algorithm. However, unlike the RF where all trees are built at the same time in parallel and each is trained independently, XGBoost builds one tree at a time sequentially and uses information from previous trees to improve subsequent trees. The idea is to correct the previous mistake made by the model, learn from it and in the next step, improve the performance. Therefore, XGBoost is greedier than the RF algorithm.

Nearest Neighbours Regression (NNr) [41] divides the data into different ‘K’ neighbourhoods (i.e., classes) using feature similarities (i.e., distances). Then, NNr assigns the new data points to the neighbourhood that is the most similar to the available ones. The NNr doesn’t perform any training, calculations, or building models until a new prediction is performed. The main advantage of NNr is that it is fast and allows users to understand and interpret the model. However, NNr struggles to adapt to highly complex relationships between features and response variables.

### Hospital based approaches for comparison

As already mentioned in "Introduction" section , the most common approach to estimating the duration of surgery is simply taking the average of previous records for the same procedure type [13]. The hospital studied in this paper also uses a similar approach by including surgeon information as well as the procedure type. At the study site, emergency surgery bookings are taken from the surgeon’s estimation of what they have entered on the booking request and estimates of emergency surgery duration can change upon findings duration the operation. Elective surgery relies on the use of a surgical estimator tool to prevent overruns and underutilisation, hence its vital to have historical information inform procedural scheduling in addition to the proceduralists’ estimates.

In this study, we had no access to the estimated procedure durations based on the above-mentioned approaches. In addition, surgeon-related information was not also available in our data, which made it almost impossible to reproduce surgery duration estimations based on the current approach at the study hospital. However, we provided two simple estimations using similar approaches to those used in the hospital: “Mean5” and “MeanAll”. The Mean5 approach takes the average of the previous 5 records of operation durations with the same procedures involved in that operation. The MeanAll approach is similar to Mean5 but takes the average of all previous records. One important note here is that, in contrast to the hospital’s approach, we used historical operations data instead of historical procedures and surgeons’ data for these methods.

### Experimental setup and evaluation measure

The prediction models were developed and implemented in Python [42] software using stat models available in the Scikit-learn library [43] and the data manipulation and visualisation were implemented in R software [44].

We investigated the models’ performance under different scenarios. First, we compared and analysed the results of the prediction models on the test data. Then, we explored the performance of the models for the single-procedure operations (i.e., operations with only one procedure) compared to the multiple-procedure operations to further examine the models in terms of accuracy. Next, the impact of explanatory variables and the Box-Cox technique applied to the response variable were examined. Finally, the proposed ML models were compared with the current methods used in hospitals.

To examine the performance of the proposed methods, we used several error statistics including Mean Absolute Error (MAE), Median Absolute Error (MdAE), Mean Absolute Percentage Error (MAPE), and R-squared value (R^2^).

The MAE calculates the average magnitude of errors between the predicted values ($F$) by a model and the actual values ($A$) for $n$ observations.

$$MAE=\frac{1}{n}\sum _{i=1}^{n}|{A}_{i}- {F}_{i}|$$

where $\sum _{i=1}^{n}|{A}_{i}- {F}_{i}|$ represents the total absolute error over $n$ observations.

The MdAE calculates the median of all absolute differences between the predicted values by a model (F) and the actual values (A) for $n$ observations. This metric is really useful since it is robust to outliers.

$$\mathrm{MdAE}=\mathrm{median}\left(\vert{\mathrm A}_1-{\mathrm F}_1\vert,\cdots,\vert{\mathrm A}_{\mathrm n}-{\mathrm F}_{\mathrm n}\vert\right)$$

The MAPE is a statistical measure that shows the absolute error between the predicted values (F) and the actual values (A) as a percentage.

$$\mathrm{MAPE}=\frac1{\mathrm n}\sum_{\mathrm i=1}^{\mathrm n}\frac{\vert{\mathrm A}_{\mathrm i}-{\mathrm F}_{\mathrm i}\vert}{{\mathrm A}_{\mathrm i}}$$

The R-squared (R^2^), also known as the coefficient of determination, indicates the percentage of variance in the response variable that can be explained by the features. R-squared calculates how well the given model fits the data. In the below formula, $\stackrel{-}{A}$ represents the average of all actual values.

$$\mathrm R^2=1-\frac{\sum_{\mathrm i=1}^{\mathrm n}{({\mathrm A}_{\mathrm i}-{\mathrm F}_{\mathrm i})}^2}{\sum_{\mathrm i=1}^{\mathrm n}{({\mathrm A}_{\mathrm i}-\overset-{\mathrm A})}^2}$$

## Results

In this section, we compare the performance of the prediction models.

Table 3 presents the results of the predictive models explained in "Predictive models" section. Among the 5 models compared, the XGBoost model obtained the best results based on all measures, followed by the NNr, RF, PR, and LR models. The XGBoost model predicted the operation duration with an MAE of 20 min and a MAPE of 32%. Also, based on MdAE, XGBoost was able to predict operation duration with a maximum error of 11 min for half of the test cases.

**Table 3:** Performance of the proposed models

Algorithms	MAE (mins)	MdAE (mins)	MAPE (%)	R^2^
LR	43	25	0.76	0.06
PR	35	17	0.55	0.25
RF	24	12	0.37	0.65
XGBoost	**20**	**11**	**0.32**	**0.82**
NNr	22	13	0.39	0.74

**Fig. 4:**
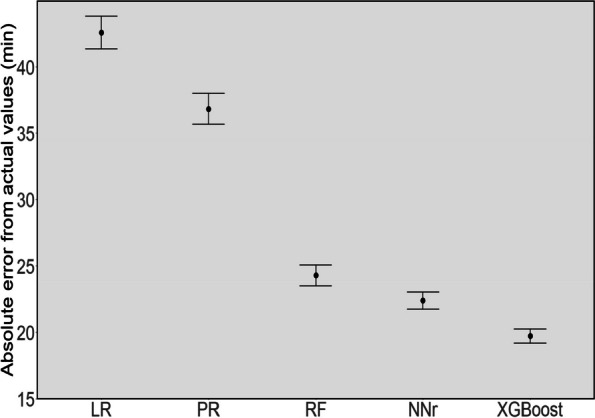
Absolute error comparison for models using a 95% confidence interval

**Fig. 5:**
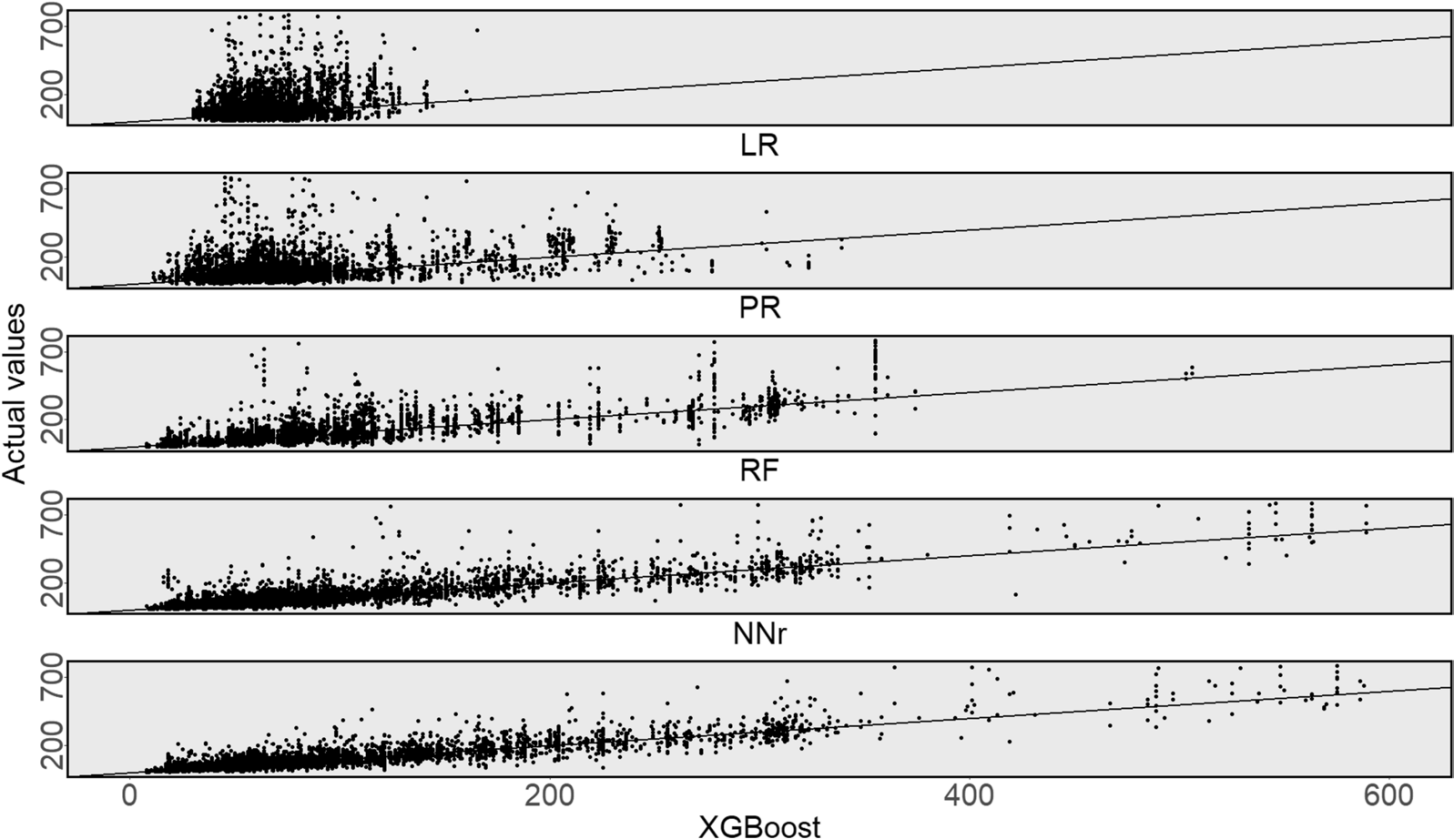
Plots of predicted operation time (horizontal axis) versus actual operation time (vertical axis); units in minutes

**Fig. 6:**
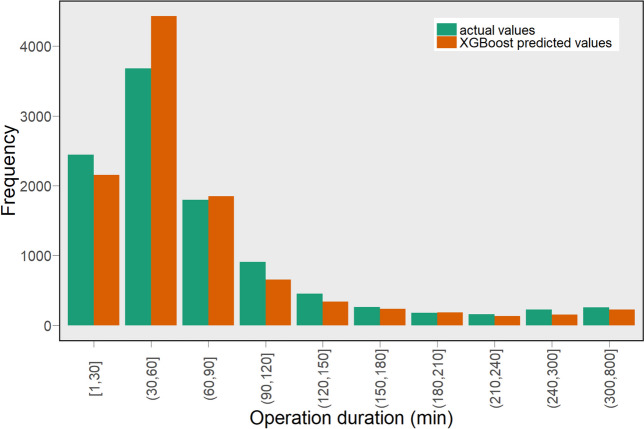
Histogram plot of the actual operation times and the predicted values by the XGBoost model

To have a better view of the performance of the models, we also present the statistical significance of the results in Fig. 4 using a 95% confidence interval plot. Figure 4 shows that the XGBoost model significantly outperformed other models. Also, we present a scatter plot of actual operation duration (vertical axis) versus predicted operation duration (horizontal axis) in Fig. 5. The scatter plot represents how well the model predicts the operation times for a different range of values. In this plot, for perfect prediction, the points should be close to the diagonal line with narrow variation around the line and minimal bias on one side of the line (to minimise under or overpredictions). As can be seen, the points of the XGBoost model predictions lie closer to the line compared to the other models and have fewer points far from the line. These findings also can be seen in Fig. 6. which shows the distribution of actual operation times and the predictions by the XGBoost model. From this figure, we can see that the XGBoost model’s predicted values are considerably close to the actual operation times in different ranges.

**Fig. 7:**
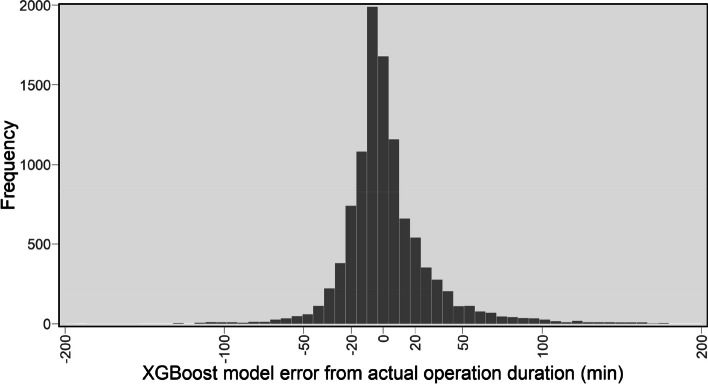
Distribution of the error of the predicted values by the XGBoost from the actual values

**Fig. 8:**
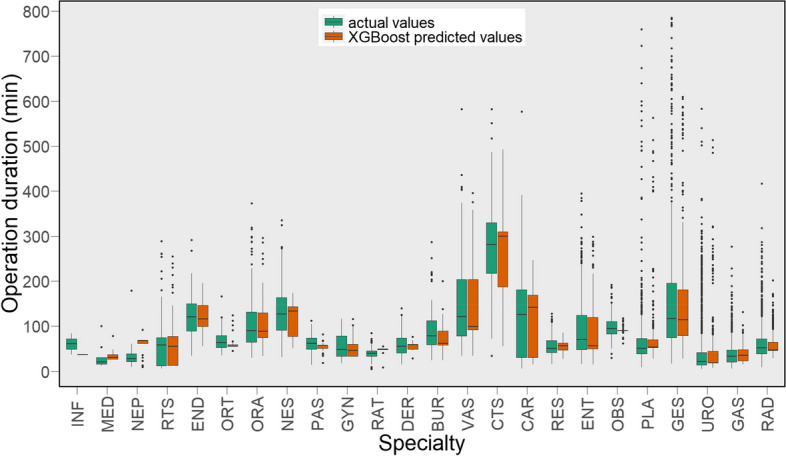
Comparison of the XGBoost model predictions versus actual operation times for different specialties

The results presented in Figs. 5 and 6 reflect the effectiveness of the XGBoost model, however, further analyses are provided to obtain a better understanding of any bias in predictions and how it performs on subgroups within the data. For example, as shown in Fig. 3, the average operation time across different specialties is notably varied, thus it would be interesting to examine the performance of the model for various specialties. To that end, we present the distribution of the actual error (i.e., not absolute error) of the XGBoost predictions in Fig. 7, and a box plot of the actual operation times versus the times predicted by the XGBoost model, separated by specialty in Fig. 8. In Fig. 7, negative and positive values represent overestimated and underestimated predictions, respectively. From this figure, we can see that the majority of the prediction errors are in the range of 25 min and are relatively unbiased. From Fig. 8 also we can see that the model performed robustly as it has a similar distribution shape to the actual values for most specialties.

For a further comparison of the models’ performance, we split the test set results for operations with a single procedure and multiple procedures. The test dataset includes 9147 operations with a single procedure with a mean operation duration of 70 min and a median of 48 min. There are 1230 operations with multiple procedures with a mean operation duration of 112 min and a median of 77 min. Table 4 shows that the XGBoost model provided better R^2^ and MAPE for multiple-procedure operations, indicating that the model for multiple-procedure operations performed as well, if not better than single-procedure operations.

**Table 4:** Performance of models for single- and multiple-procedure operations

Algorithm	Single Procedure				Multiple Procedures			
	MAE (mins)	MdAE (mins)	MAPE (%)	R^2^	MAE (mins)	MdAE (mins)	MAPE (%)	R^2^
LR	40	24	0.78	0.06	63	36	0.66	-0.04
PR	32	17	0.55	0.28	55	26	0.51	0.06
RF	22	12	0.38	0.65	35	17	0.31	0.60
XGBoost	**19**	**11**	**0.33**	**0.81**	**27**	**15**	**0.27**	**0.82**
NNr	21	13	0.39	0.77	34	16	0.32	0.63

To examine the impact of patient-based features in predicting operation duration, we created two new sets of models: model_O and model_P. The model_O models used only operation-based features (specialty, number of procedures, and procedure combination), while the model_P models used only patient-based features (CCI and ASA). Table 5 shows the performance of these models compared to the original models built on the combination of both sets of features (designated here as model_OP). It is observed that model_O generally outperforms model_P (a minimum of 40% difference in terms of MAPE for three models: RF, XGBoost, and NNr). This indicates the importance of operation-based features for surgery duration prediction. Also, a statistical test between model_O and model_OP showed that the majority of our predictive models are statistically similar, except for LR and PR, which indicates that adding patient-based features to model_O just marginally, not significantly, impacted the models’ performance.

**Table 5:** Comparison of the models with different feature settings

Algorithm	Model_P				Model_O				model_OP			
	MAE (mins)	MdAE (mins)	MAPE (%)	R^2^	MAE (mins)	MdAE (mins)	MAPE (%)	R^2^	MAE (mins)	MdAE (mins)	MAPE (%)	R^2^
LR	44	26	0.80	0.00	44	26	0.76	0.01	43	25	0.76	0.06
PR	44	25	0.78	0.01	40	21	0.64	0.07	37	19	0.58	0.20
RF	45	25	0.78	0.02	24	12	0.37	0.62	24	12	0.37	0.63
XGBoost	44	25	0.78	0.01	**20**	12	0.33	0.81	**20**	**11**	**0.32**	**0.82**
NNr	46	30	1.00	0.02	23	14	0.39	0.76	22	13	0.39	0.74

To further quantify the importance of operation-based and patient-based features we conducted the permutation importance technique using the model_OP. Since ML models function as “black boxes” [45] and often lack explainability, this technique is important for the development of prediction models since it provides insight into the data and makes it easier to understand and explain the model output [46]. This technique assigns a score to features based on how useful they are at predicting a response variable. From Table 6, the operation-based features were largely more important than the patient-based features.

**Table 6:** The feature importance score

Features	Importance score
Specialty	0.58
Procedure combination	0.23
Number of procedures	0.11
ASA score	0.06
CCI	0.02

As already discussed in  "Data sources" section , we used the Box-Cox technique on the response variable (operation duration) since its distribution is positively skewed (i.e., the distribution tail is more pronounced to its right). In this section, we compared the results of the developed predictive models with the original operation durations (Org_OD) and the models developed after using Box-Cox methods (Box-Cox_OD). The results are presented in Table 7 and show that using the Box-Cox method on the response variable improves MAPE by 6% for the XGBoost and NNr models and by more than 10% for the other three models. Also, the R^2^ of all models except LR improves by between 1% and 3% which confirms that for this dataset, using the Box-Cox method on the response variable helps models represent the data better.

**Table 7:** Comparison of predictive performance with and without the Box-Cox method

Algorithm	Org_OD				Box-Cox_OD			
	MAE (mins)	MdAE (mins)	MAPE (%)	R^2^	MAE (mins)	MdAE (mins)	MAPE (%)	R^2^
LR	48	35	1.17	0.12	43	25	0.76	0.06
PR	45	32	1.05	0.19	37	19	0.58	0.20
RF	27	16	0.50	0.62	24	12	0.37	0.63
XGBoost	21	13	0.38	0.81	**20**	**11**	**0.32**	**0.82**
NNr	24	14	0.45	0.71	22	13	0.39	0.74

The results comparing the performance of the developed prediction model to common hospital approaches described in "Hospital based approaches for comparison" section (Mean5 and MeanAll) are presented in Table 8. It is observed that the XGBoost model outperforms both Mean5 and MeanAll, delivering a reduction in MAPE of 6%. The standard error (SE) and the 95% confidence intervals (95% CIs) of the developed XGBoost and the common hospital methods are also presented in Table 8. As can be seen, the XGBoost showed better performance than both Mean5 and MeanAll, but the difference in performance between XGBoost and MeanAll is not statistically significant.

**Table 8:** Comparison of proposed models with some possible current hospital methods

Algorithms	MAE (mins)	MdAE (mins)	MAPE (%)	SE	95% CIs
XGBoost	**20**	**11**	**0.32**	**0.271**	**(19.11, 20.18)**
Mean5	21	13	0.38	0.282	(20.57, 21.68)
MeanAll	21	12	0.38	0.280	(20.07, 21.17)

## Discussion

### The models’ performance

 In "Results" section, we evaluated the performance of the ML models and showed that the XGBoost model outperformed other ML models compared, as shown in Table 3; Fig. 4. The performance of the XGBoost model was also further analysed to obtain a better understanding of any bias and predictions on subgroups within the data. While we observed more overestimation than underestimation (around 11%, see Fig. 7), the total volume of underestimation is higher, 17% to be exact. This can be attributed to the large number of records whose durations are outliers (see Fig. 8) compared to other records for which the models normally predict the operation duration to be less than the actual cases. Besides, as shown in Fig. 3, the average operation times of the specialties are varied and the performance of the model for different specialties reveals some interesting findings (see Fig. 8). From this figure, first, some specialties have a large number of outliers (i.e., the dot points outside the whiskers) including URO and GES, which are among the most common specialties for elective surgeries in the dataset. Second, for almost all specialties, the median of prediction values is considerably close, if not the same, to the median of the actual value*s. Thirdly, the prediction values for several specialties such as RAD, GAS, and GES are largely similar to the actual ones, not only in terms of median values but also first and third quartiles. Given that these are the most common specialties (as shown in Fig. 2), we can conclude that more records help the model learn better and find a better pattern for prediction. Fourthly, even for specialties with a wide range of operation duration (i.e., the boxes are long) such as CAR and GES, the box plot of the predicted values has a similar distribution shape to the actual values, which indicates the robustness of the model. There are considerable deviations between the actual and predicted values for a number of specialties, including NEP and RAT, which warrants further investigation.

### Impact and selection of explanatory variables

In this study, we used two categories of features (i.e., explanatory variables): operation-based features and patient-based features. The effectiveness of operation-based features for predicting surgery duration is also extensively discussed in the literature [30, 32, 44, 45]. However, there are inconsistencies about the importance of patient-based features. Eijkemans et al., [30] showed that patient-based features significantly influence the performance of the models. Similarly, Bartek et al., [32] indicated that patient-based features such as age and patient class (e.g., inpatient or outpatient) are among the top features for model development. On the other hand, Edelman et al., [14] used age and the ASA score as patient-based features in their model and pointed out that these two features are not a factor in improving the model’s accuracy. Combes et al., [45] analysed different factors affecting operation durations in the endoscopy department and showed that they are not dependent on patient-based features. Our findings also showed that the developed models for surgery duration prediction substantially relied on operation-based features rather than patient-based ones (as shown in Table 5), and, based on the permutation importance technique calculations, the “specialty” was the most important feature, and the patient-based features were comparatively low importance.

### Single- and multiple-procedure operations

Most existing research studies focus on predicting procedure-level durations as opposed to the entire operation duration (note, an operation may consist of a single or multiple procedures). However, operations include some extra workflow steps besides the underlying procedure times, which make it more challenging to be predicted. In our dataset, for operations with a single procedure, on average, 64% of the operation duration was attributed to the procedure time and the other 36% of the operation duration associated to other tasks. This ratio may differ for each procedure or specialty. The difficulty of using procedure times for estimating the entire operation time is significantly greater for operations with multiple procedures. This is because multiple procedures are not necessarily performed sequentially and may happen at the same time. The prediction models in this study yield lower MAE and MdAE but higher MAPE for single-procedure operations than multiple-procedure operations. This could be interpreted by the fact that the average and median of single-procedure operations are noticeably less than those of multiple-procedure operations, by 42 and 29 min, respectively.

### Comparison with state-of-the-art methods

As discussed in "Introduction" section, multiple models have been developed for predicting either procedure or operation durations [47]. Due to the differences in datasets, features, and experimental design, it is hard, if not impossible, to directly compare the results of the proposed models with those in the literature. However, the following presents some methodological comparisons that highlight the novelty and the effectiveness of the models developed in this study.

ShahabiKargar et al. [8] developed 3 different ML models for predicting procedure duration and found that RF obtained the best results with a MAPE of 0.68 and an R^2^ of 0.65. Kayış et al. [16] studied two years of elective surgeries in a children’s hospital, comprising 8096 records. They used statistical methods to adjust the surgeon’s estimation for predicting the procedure time which obtained an R^2^ of 0.69 and a MAE of 38 min. Master et al., [48] provided decision tree methods for predicting surgery time (defined as the time surgeons enter the OR to leave the OR) which yielded an R^2^ of 0.61. They predicted the duration that surgeons rather than patients spend in the OR which is not relevant to OR utilisation. The main common limitation of these three studies is that they focus on predicting procedure times rather than the actual operation duration.

Devi et al., [19] proposed an Artificial Neural Network (ANN)-based approach for predicting operation duration. The scope of their study was limited to the operations performed within an ophthalmology department and for only three different procedures. In addition, their data was limited to only 100 records. Eijkemans et al., [30] proposed linear models for predicting operation duration using a wide range of potential features (e.g., surgical team, surgeon, and patient) which obtained an R^2^ of 0.79. However, they focused on operations undertaken only within the general surgery department. Bodenstedt et al., [49] proposed ANN models for procedure duration prediction that achieved a MAPE of 0.37 (cf. MAPE of 0.32 for XGBoost in our study). However, their approach was limited to only laparoscopic operations. Garside et al., [50] proposed an XGBoost model for the prediction of operation duration for colorectal and spinal surgeries with an MAE of 37 min (cf. MAE of 20 min for XGBoost in our study). Edelman et al. [14] developed multiple models for predicting the total surgical procedure time (defined as total surgical time and anaesthesia time) and reported that the best prediction model estimated the actual values with an MAE of 31 min. They used the estimated surgery time as a feature that was obtained by simply subtracting 20 min from the actual total procedure time. Tuwatananurak et al., [51] developed an ML model for predicting operation duration that achieved a MdAE of 20 min (cf. MdAE of 11 min for XGBoost in our study). They collected only three months of data for model development and also used features that may not be available several days before the surgery, i.e., at the time of scheduling (e.g., cancellation of prior operations, and the time that operations started). Bartek et al., [32] developed an ML model for predicting operation duration that achieved an R^2^ of 0.78 and a MAPE of 28%. However, they trained the data on only 12 selected specialties. A common limitation across the above-mentioned studies was that they focused only on a limited number of specialties or procedures.

While not directly comparable, our approach for operation duration prediction resulted in better performance metrics than the ones in the above-mentioned related work. In addition, we also addressed some of the limitations that exist in the literature; Firstly, we predicted the operation duration, not the procedure duration or surgery duration (i.e., the time between the surgeon entering the room and the surgeon leaving [48]). Secondly, we used a dataset collected over four years, including over 50,000 elective operations. Having a large dataset allows us to provide a comprehensive investigation of potential factors affecting the ML models for the operation duration prediction task. Thirdly, we did not restrict our models to a single or selected subset of specialties, our dataset includes over 26 specialties and 500 procedure combinations. This helps the clinical administrative team, in reality, manage ORs with a broader range of specialties. Fourthly, we used features that are all available preoperatively, i.e., several days before the surgery, which enables our approach to be deployed in the scheduling environment. Finally, our approach was able to consistently predict operation duration for both single-procedure operations as well as the more challenging multiple-procedure operations.

### Comparison with the current hospital methods

Comparing the XGBoost model and the MeanAll approach to actual operation duration indicated that the XGBoost model achieved 6854 min less of total absolute error over 1 year. It may be argued that the amounts gained in absolute minutes can be considered not significant, however, this can be translated to meaningful gains for the hospital and OR management in the long-term horizon. Knowing the fact that the average cost for 1 min of OR time is estimated to be between 22 and 133 USD [52], this improvement may translate to a significant reduction of costs.

### Limitations

The data used in this study was collected from an administrative database providing information about elective and emergency surgeries. The data used in this study did not contain surgeon-related information, including surgeon names, surgeon’s estimation of the operations and the number of anaesthetists and nurses involved in the operations. This missing information limited both our model development and our comparisons with the current hospital methods. Also, being a single-site study, our data was obtained from one hospital, and hence, was limited to a particular demographic area, a distinct pattern of surgical demand, and elective case-booking techniques. Considering the mentioned limitations, it should be noted that the broad generalisation and application of our findings should be done with caution to non-elective surgeries or other hospitals.

## Conclusions

In this study, we developed and validated predictive models for estimating surgery duration, using data related to four years of elective surgeries undertaken in a large metropolitan hospital in Australia. To make the model, we considered several realistic scenarios, predicting the operation duration rather than procedure duration, considered operations with both single and multiple procedures, considered features known at the time of creating OR lists, and considered all common specialties and procedures. The XGBoost predictive model yielded the best results with R^2^ of 82% and MdAE of 11 min, reducing the total absolute error by 6854 min (i.e., about 114 h) over 1 year compared to current hospital methods. For future work, there is still room for model improvement, particularly through incorporating information related to staff (surgeons and nurses) and the actual surgeon’s estimation of the operations. Also, other studies including different types of datasets need to be performed to determine the generalisability of these results. The results of the case study in this paper show that delivering a significant efficiency improvement required further developments, e.g., additional efforts are needed towards improving the standardisation of the data capture and explainability and interoperability of the ML algorithms and generalisability across multiple sites.

### Supplementary Information

**Additional file 1.**

## Data Availability

Data analysed in this study is unable to be shared due to legislative and review committee requirements. The original data are available from Western Australia’s Department of Health subject to appropriate governance and ethical approvals. Data can be requested from Health Support Services, within the Government of Western Australia (SHaRESupport@health.wa.gov.au, www.hss.health.wa.gov.au).

## References

[1] Macario A, Dexter F (1999). Estimating the duration of a case when the surgeon has not recently scheduled the procedure at the surgical suite. Anesth Analgesia.

[2] Childers CP, Maggard-Gibbons M (2018). Understanding costs of care in the operating room. JAMA Surg.

[3] Schofield WN (2005). Cancellation of operations on the day of intended surgery at a major Australian referral hospital. Med J Aust.

[4] Thompson TP, Brown HN (2002). Turnover of licensed nurses in skilled nursing facilities. Nurs Econ.

[5] Strachota E (2003). Reasons registered nurses leave or change employment status. JONA: The Journal of Nursing Administration.

[6] Armoeyan M, Aarabi A, Akbari L (2021). The effects of surgery cancellation on patients, families, and staff: a prospective cross-sectional study. J PeriAnesthesia Nurs.

[7] Soh KW (2020). Case study of the prediction of elective surgery durations in a New Zealand teaching hospital. Int J Health Plann Manag.

[8] ShahabiKargar Z (2014). Predicting procedure duration to improve scheduling of elective surgery. Pacific Rim International Conference on Artificial Intelligence.

[9] Wang Z, Dexter F (2022). More accurate, unbiased predictions of operating room times increase labor productivity with the same staff scheduling provided allocated hours are increased. Perioperative Care and Operating Room Management.

[10] Adams T, O’Sullivan M, Walker C (2023). Surgical procedure prediction using medical ontological information. Comput Methods Programs Biomed.

[11] Laskin DM, Abubaker AO, Strauss RA (2013). Accuracy of predicting the duration of a surgical operation. J Oral Maxillofac Surg.

[12] May JH (2011). The surgical scheduling problem: current research and future opportunities. Prod Oper Manage.

[13] Zhou J (1999). Relying solely on historical surgical times to estimate accurately future surgical times is unlikely to reduce the average length of time cases finish late. J Clin Anesth.

[14] Edelman ER (2017). Improving the prediction of total surgical procedure time using linear regression modeling. Front Med.

[15] Cassera MA (2009). Surgical time independently affected by surgical team size. Am J Surg.

[16] Kayış E (2015). A robust estimation model for surgery durations with temporal, operational, and Surgery team effects. Health Care Manag Sci.

[17] Strum DP (2000). Surgeon and type of anesthesia predict variability in surgical procedure times. J Am Soc Anesthesiologists.

[18] Wang Z, Dexter F, Zenios SA (2020). Caseload is increased by resequencing cases before and on the day of surgery at ambulatory surgery centers where initial patient recovery is in operating rooms and cleanup times are longer than typical. J Clin Anesth.

[19] Devi SP, Rao KS, Sangeetha SS (2012). Prediction of surgery times and scheduling of operation theaters in optholmology department. J Med Syst.

[20] Joustra P, Meester R, van Ophem H (2013). Can statisticians beat surgeons at the planning of operations?. Empirical Economics.

[21] Curtis N (2019). Artificial neural network individualised prediction of time to colorectal cancer surgery. Gastroenterol Res Pract.

[22] Huber M, Kurz C, Leidl R (2019). Predicting patient-reported outcomes following hip and knee replacement surgery using supervised machine learning. BMC Med Inf Decis Mak.

[23] Maillo J (2017). kNN-IS: an iterative spark-based design of the k-Nearest neighbors classifier for big data. Knowl Based Syst.

[24] Abbou B (2022). Optimizing operation room utilization—A prediction model. Big Data and Cognitive Computing.

[25] Babayoff O (2022). Surgery duration: optimized prediction and causality analysis. PLoS ONE.

[26] Dean A (2022). Quantile regression forests for individualized surgery scheduling. Health Care Manag Sci.

[27] Master N (2017). Improving predictions of pediatric surgical durations with supervised learning. Int J Data Sci Analytics.

[28] Bland J, Altman D, Rohlf F (2013). Defence of logarithmic transformations. Stat Med.

[29] Strum DP, May JH, Vargas LG (2000). Modeling the uncertainty of surgical procedure times: comparison of log-normal and normal models. J Am Soc Anesthesiologists.

[30] Eijkemans MJ (2010). Predicting the unpredictable: a new prediction model for operating room times using individual characteristics and the surgeon’s estimate. J Am Soc Anesthesiologists.

[31] Marimuthu S (2022). Preferring Box-Cox transformation, instead of log transformation to convert skewed distribution of outcomes to normal in medical research. Clin Epidemiol Global Health.

[32] Bartek MA (2019). Improving operating room efficiency: machine learning approach to predict case-time duration. J Am Coll Surg.

[33] Haugan K, Klaksvik J, Foss OA (2021). 30-day mortality in patients after hip fracture surgery: a comparison of the Charlson comorbidity index score and ASA score used in two prediction models. Injury.

[34] Gasparini A (2018). Comorbidity: an R package for computing comorbidity scores. J Open Source Softw.

[35] Harris MB (2010). Mortality in elderly patients after cervical spine fractures. J Bone Joint Surg Am Vol.

[36] Zou KH, Tuncali K, Silverman SG (2003). Correlation and simple linear regression. Radiology.

[37] Stigler SM (1974). Gergonne’s 1815 paper on the design and analysis of polynomial regression experiments. Hist Math.

[38] Forests R (2001). By Leo Breiman. Mach Learn.

[39] Li B (1984). Classification and regression trees (CART). Biometrics.

[40] Chen T, Guestrin C. Xgboost: a scalable tree boosting system. In: Proceedings of the 22nd acm sigkdd international conference on knowledge discovery and data mining. 2016. p. 785–94.

[41] Fix E, Hodges JL. Discriminatory analysis: nonparametric discrimination: small sample performance. Randolph Field TX Air University USAF School of Aviation Medicine; 1952.

[42] Sanner MF (1999). Python: a programming language for software integration and development. J Mol Graph Model.

[43] Pedregosa F (2011). Scikit-learn: machine learning in Python. J Mach Learn Res.

[44] R Core Team, R and others. R: a language and environment for statistical computing. Vienna; 2013.

[45] Beam AL, Kohane IS (2018). Big data and machine learning in health care. JAMA.

[46] Kuhn M, Johnson K. Applied predictive modeling, vol. 26. Springer; 2013.

[47] Dexter F. *Predicting operating room task durations*. 2023 July, 5 2023]; Available from: https://franklindexter.net/bibliography_PredictingDuration.htm.

[48] Master N (2017). Improving predictions of pediatric surgical durations with supervised learning. Int J Data Sci Analytics.

[49] Bodenstedt S (2019). Prediction of laparoscopic procedure duration using unlabeled, multimodal sensor data. Int J Comput Assist Radiol Surg.

[50] Garside N (2021). CPT to RVU conversion improves model performance in the prediction of surgical case length. Sci Rep.

[51] Tuwatananurak JP (2019). Machine learning can improve estimation of surgical case duration: a pilot study. J Med Syst.

[52] Macario A (2010). What does one minute of operating room time cost?. J Clin Anesth.

