# Supplementary material for: Improving preoperative prediction of surgery duration
Source: BMC Health Serv Res. 2023 Dec 2;23:1343. doi: 10.1186/s12913-023-10264-6 (PMC10693694; doi:10.1186/s12913-023-10264-6)
Supplement: Supplementary file 1 — Additional file 1. [file 12913_2023_10264_MOESM1_ESM.docx]

**Supplementary data**

As mentioned in the paper, we conducted a sensitivity analysis to explore alternative scenarios for missing data. We created multiple datasets after applying multiple imputation procedures such as mode, median, last observation comes forward (LOCF) and KNN. We also created another dataset in which all missing values were filtered out, i.e., only keeping the complete records (i.e., “Removing all NAs”). We compared the results of the two high-performing ML models in the papers (i.e., XGBoost and NNr) for each scenario based on the MAPE and the results are shown in the table below.

|  | **algorithms** | |
| --- | --- | --- |
| **Method** | **XGBoost** | **NNr** |
| KNN | **0.82** | 0.74 |
| LOCF | 0.81 | 0.75 |
| Median | **0.82** | 0.75 |
| Mode | **0.82** | 0.75 |
| Removing all NAs | **0.82** | 0.76 |

As can been seen, both models perform almost equally well on all datasets with various imputation procedures. Besides, the fact that the results after imputation of the missing data for the ASA score were very similar to the complete records indicates that imputing the large number of missing values for the ASA score variable has limited influence on the outcomes.
